# Sodium butyrate restored TRESK current controlling neuronal hyperexcitability in a mouse model of oxaliplatin-induced peripheral neuropathic pain

**DOI:** 10.1016/j.neurot.2024.e00481

**Published:** 2024-11-13

**Authors:** Idy H.T. Ho, Yidan Zou, Kele Luo, Fenfen Qin, Yanjun Jiang, Qian Li, Tingting Jin, Xinyi Zhang, Huarong Chen, Likai Tan, Lin Zhang, Tony Gin, William K.K. Wu, Matthew T.V. Chan, Changyu Jiang, Xiaodong Liu

**Affiliations:** a Department of Anaesthesia and Intensive Care, Faculty of Medicine, The Chinese University of Hong Kong, Hong Kong Special Administrative Region, China; b Peter Hung Pain Research Institute, Li Ka Shing Institute of Health Sciences, The Chinese University of Hong Kong, Hong Kong Special Administrative Region, China; c The Chinese University of Hong Kong, Shenzhen, China; d Institute of Digestive Disease, Li Ka Shing Institute of Health Sciences, The Chinese University of Hong Kong, Hong Kong Special Administrative Region, China; e State Key Laboratory of Digestive Disease, The Chinese University of Hong Kong, Hong Kong Special Administrative Region, China; f Microbiota I Centre (MagIC), The Chinese University of Hong Kong, Hong Kong Special Administrative Region, China; g Department of Medicine and Therapeutics, Faculty of Medicine, The Chinese University of Hong Kong, Hong Kong Special Administrative Region, China; h Department of Pain Medicine and Shenzhen Municipal Key Laboratory for Pain Medicine, The 6th Affiliated Hospital of Shenzhen University Medical School, Shenzhen, China

**Keywords:** Chemotherapy-induced peripheral neuropathy, Butyrate, Epigenetics, Histone deacetylase inhibition, Dorsal root ganglion

## Abstract

Chemotherapy-induced peripheral neuropathy (CIPN) and its related pain are common challenges for patients receiving oxaliplatin chemotherapy. Oxaliplatin accumulation in dorsal root ganglion (DRGs) is known to impair gene transcription by epigenetic dysregulation. We hypothesized that sodium butyrate, a pro-resolution short-chain fatty acid, inhibited histone acetylation in DRGs and abolished K^+^ channel dysregulation-induced neuronal hyperexcitability after oxaliplatin treatment. Mechanical allodynia and cold hyperalgesia of mice receiving an accumulation of 15 ​mg/kg oxaliplatin, with or without intraperitoneal sodium butyrate supplementation, were assessed using von Frey test and acetone evaporation test. Differential expressions of histone deacetylases (HDACs) and pain-related K^+^ channels were quantified with rt-qPCR and protein assays. Immunofluorescence assays of histone acetylation at H3K9/14 were performed in primary DRG cultures treated with sodium butyrate. Current clamp recording of action potentials and persistent outward current of Twik-related-spinal cord K^+^ (TRESK) channel were recorded in DRG neurons with small diameters extract. Accompanied by mechanical allodynia and cold hyperalgesia, HDAC1 was upregulated in mice receiving oxaliplatin treatment. Sodium butyrate enhanced global histone acetylation at H3K9/14 in DRG neurons. *In vivo* sodium butyrate supplementation restored oxaliplatin-induced *Kcnj9* and *Kcnk18* expression and pain-related behaviors in mice for at least 14 days. Oxaliplatin-induced increase in action potentials frequencies and decrease in magnitudes of KCNK18-related current were reversed in mice receiving sodium butyrate supplementation. This study suggests that sodium butyrate was a useful agent to relieve oxaliplatin-mediated neuropathic pain.

## Introduction

Peripheral neuropathy is one of the most common reasons for limiting chemotherapy administration in cancer patients. In particular, oxaliplatin, a third-generation platinum-based chemotherapeutic agent against advanced colorectal cancer (CRC), induces acute chemotherapy-induced peripheral neuropathy (CIPN) in up to 92 ​% of patients [1,2] and chronic CIPN in approximately 70 ​% of patients following prolonged treatment courses [3]. Approximately 60 ​% of patients experienced persistent neuropathic symptoms, including paranesthesia, hypoesthesia and dysesthesia in the extremities, that may interfere with functional activities between and after cycles of oxaliplatin treatment [4]. The persistence of oxaliplatin-dependent neurotoxicity and its side effects often resulted in prolongation of infusion time, dose reduction, treatment delay or cassation and a lower quality of life [4,5]. The number of clinically approved analgesics against oxaliplatin-induced CIPN is very limited. Since most alternatives are of low efficacy, there is a massive medical demand to discover novel drug candidates that can prevent the development of CPIN.

Oxaliplatin accumulation in the dorsal root ganglion (DRGs) is considered a crucial mechanism of chronic oxaliplatin-associated CIPN [6]. Oxaliplatin induced cell death and inhibited neurite outgrowth in dose-dependent manners in the DRG neurons [7,8]. We recently highlighted neuroinflammation may take an important role in oxaliplatin-induced CIPN [9]. Although the exact pathophysiology remains uncertain, oxaliplatin-induced nucleolar damage, mitochondrial dysfunction, oxidative stress and ion channel expression remodeling may underlie oxaliplatin-induced neuropathy and pain hypersensitivity [6,[9], [10], [11]].

Epigenetic modification is highlighted as a basis of multiple cellular responses to chemotherapy. Histone deacetylase (HDAC) enzymes remove acetylation from protein lysine residues in the N-terminal of the core histones, leading to a more closed chromatin architecture and a repression of gene expression [12]. In neurodegenerative diseases, histone acetylation homeostasis shifts toward hypoacetylation, while application of HDAC inhibitors (HDACIs) leads to neuroprotection [13]. A single dose of oxaliplatin enhanced HDAC3 expression in mice, while MS-275, a class I HDACI prevented the oxaliplatin-induced acute and chronic pain symptoms [14,15]. Recent reports further reinforced analgesic-like effects of the HDACIs treatments in models of osteoarthritis-induced chronic inflammatory pain, peripheral nerve injury-induced neuropathic pain, chemotherapy-induced and bone cancer-induced pain [[16], [17], [18], [19], [20], [21]].

Sodium butyrate (NaB) is one of the effective HDACIs with anti-inflammatory effects and neuroprotective effects. NaB and butyrate-based drugs, such as N-(1-carbamoyl-2-phenyl-ethyl) butyramide, showed analgesic-like effects in fibromyalgia, migraine, and preclinical models of pain induced by chronic-constriction injury, irritable bowel syndrome, bone cancer and after exposure to paclitaxel and long-term morphine administration [[22], [23], [24], [25], [26], [27], [28]]. A clinical trial further demonstrated NaB reduced abdominal pain in patients with irritable bowel syndrome [29]. While the HDAC inhibitory and analgesic-like properties of NaB were independently demonstrated in previous studies [12,[22], [23], [24], [25], [26], [27], [28],^30^], it remains uncertain on the relationship between NaB pain-relieving effects and HDAC inhibition in DRG tissues.

In this study, we hypothesized that supplementation of NaB inhibited HDAC hyperactivity. By restoring gene expression in DRG neurons, NaB alleviated CIPN-related pain in a mouse model of oxaliplatin-induced peripheral neuropathy. The study envisaged that NaB served as a novel drug candidate against oxaliplatin-induced CIPN by directly targeting the DRG tissues.

## Materials and Methods

### Animals

C57BL/6 mice (5–8 weeks, male and female) were provided by the Laboratory Animal Services Centre, the Chinese University of Hong Kong and the 6th Affiliated Hospital of Shenzhen University Medical School. The animals were housed in groups per cage with animal chow and water ad libitum on a 12 ​h light/dark cycle at 23 ​± ​2 ​°C. All experimental procedures were approved by the Animal Experimentation Ethics Committee, The Chinese University of Hong Kong or the 6th Affiliated Hospital of Shenzhen University Medical School.

### Oxaliplatin and sodium butyrate administration

**Fig. 1:**
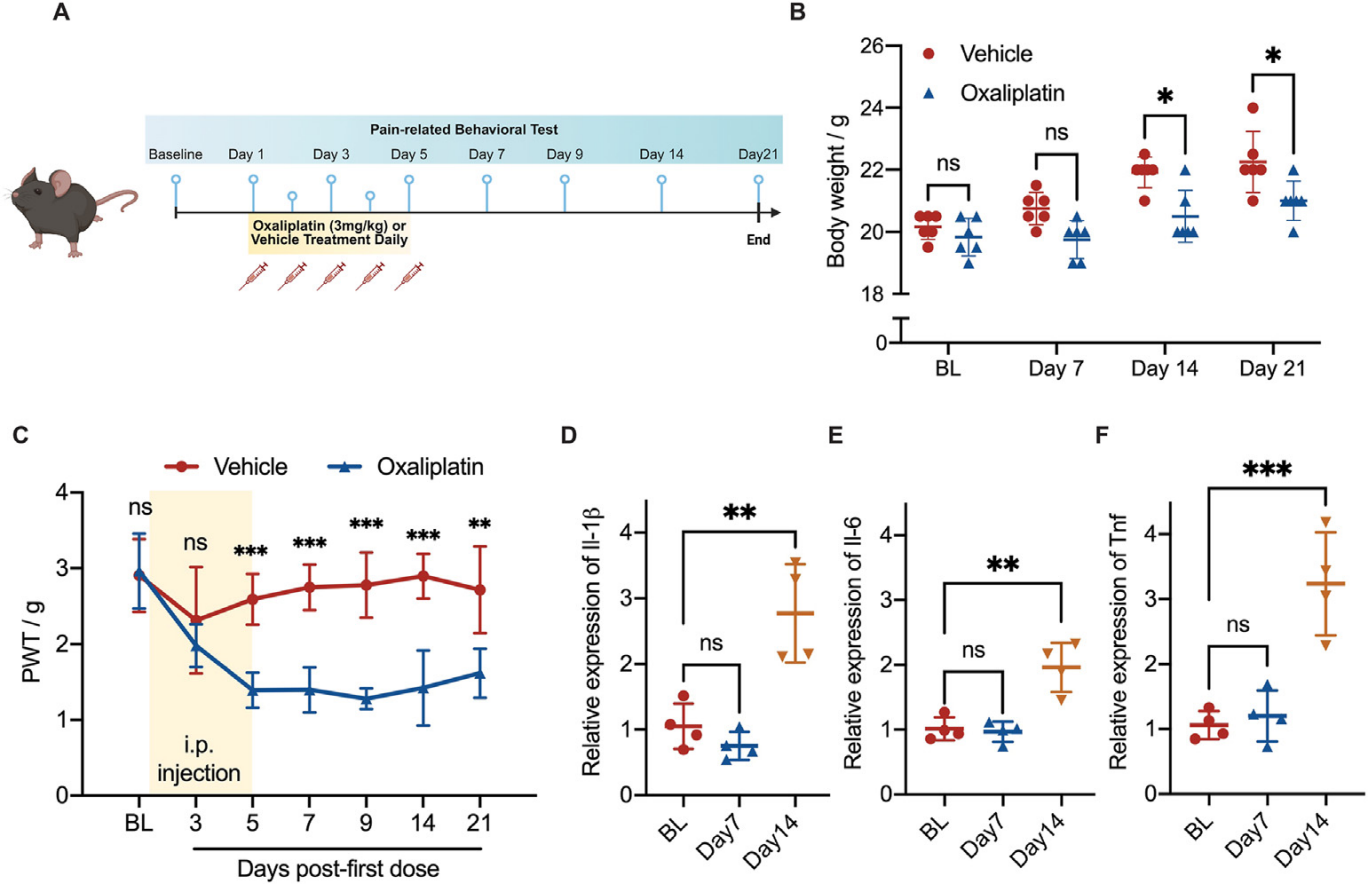
**Oxaliplatin-induced peripheral neuropathy induced mechanical allodynia in mice.** **A**, An illustration of the experimental timeframe for pain-related behavioral tests. Male mice were intraperitoneally injected with 3 ​mg/kg oxaliplatin (Oxa) or vehicle (Veh) for 5 consecutive days (Day 1–5, cumulative dose: 15 ​mg/kg, i.p.). Pain-related behavior was tested with electronic von Frey on baseline (BL), Day 3, 5, 7, 9, 14 and 21 post-first injection. **B**, Body weight of male mice received oxaliplatin or vehicle injections. **C**, Paw withdrawal thresholds (PWT) of male mice after oxaliplatin or vehicle injections. Two-way ANOVA followed by Tukey's multiple comparisons test, *n* ​= ​6 per group. ns, non-significant, ∗∗*p* ​< ​0.01, ∗∗∗*p* ​< ​0.001. **D-F**, Cytokine production (*Il-6*, *Il-1β* and *Tnf*) in L4-L5 DRG of male mice at BL, Day 7 and 14 post-injection were examined with rt-qPCR. One-way ANOVA followed by Tukey's multiple comparisons test, *n* ​= ​4 per group. ns, non-significant, ∗∗*p* ​< ​0.01, ∗∗∗*p* ​< ​0.001. All data were reported as the mean ​± ​S.D.

To establish oxaliplatin-induced mice model of CIPN, oxaliplatin (Dalian Meilun Biology Technology Co. ltd) was dissolved in 5 ​% glucose in PBS and injected intraperitoneally at 3 ​mg/kg in a volume of 100 ​μL daily for 5 consecutive days. Sham animals received 5 ​% glucose in PBS without oxaliplatin (Fig. 1A).

**Fig. 2:**
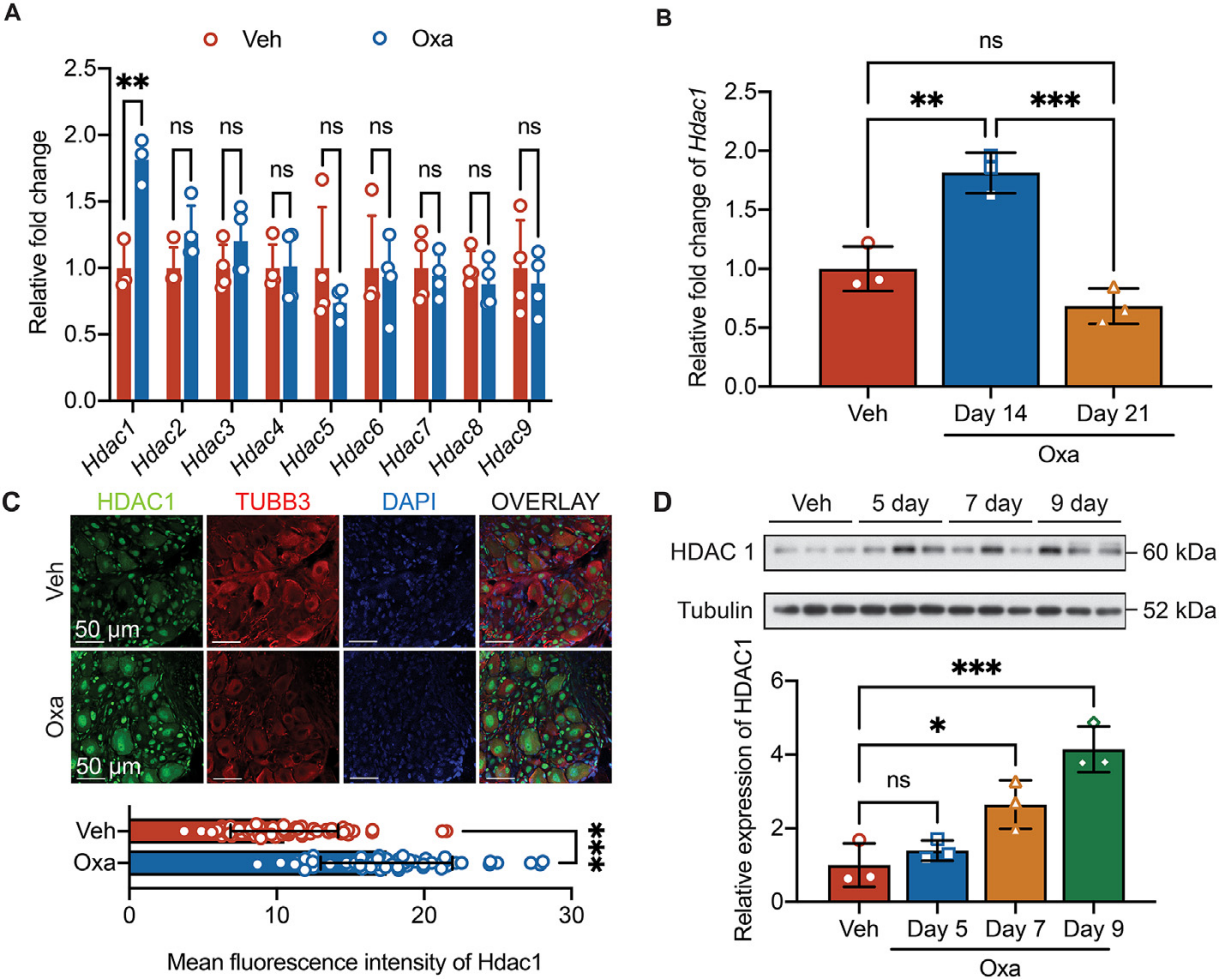
**Oxaliplatin-induced enhanced expression of HDAC1 in DRG tissues.** **A**, Expression levels of HDAC subtypes in DRG tissues of male mice receiving oxaliplatin (Oxa) or vehicle (Veh) on day 14 post-injection. Unpaired *t*-test comparing Veh and Oxa group per gene, *n* ​= ​3–4 independent mice per group. ns, non-significant, ∗∗*p* ​< ​0.01. **B**, Expression level of Hdac1 in DRG tissues of Veh or Oxa group along the development of oxaliplatin-mediated CIPN-induced pain hypersensitivity. One-way ANOVA followed by Tukey's multiple comparisons test, *n* ​= ​3 independent male mice per group. ns, non-significant, ∗∗*p* ​< ​0.01, ∗∗∗*p* ​< ​0.001. **C**, *Top*, representative immunofluorescence of HDAC1 (Green), *β*III Tubulin (TUBB3, Red) and DAPI (Blue) in L3-L6 DRG tissues of Oxa or Veh group. *Bottom*, Quantification of mean fluorescence intensity of HDAC1 in nuclei of L3-L6 DRG neurons. Unpaired *t*-test, *n* ​= ​60 from 3 male mice per group. ∗∗∗*p* ​< ​0.001. **D**, *Top*, representative Western blot of HDAC1 in L3-L6 DRG tissues of Oxa or Veh group collected 5,7 or 9 days after first injection. *Bottom*, densitometry quantification of HDAC1 protein content normalized with tubulin. One-way ANOVA followed by Tukey's multiple comparisons test, *n* ​= ​3 male mice per group. ns, non-significant, ∗*p* ​< ​0.05, ∗∗∗*p* ​< ​0.001.

**Fig. 3:**
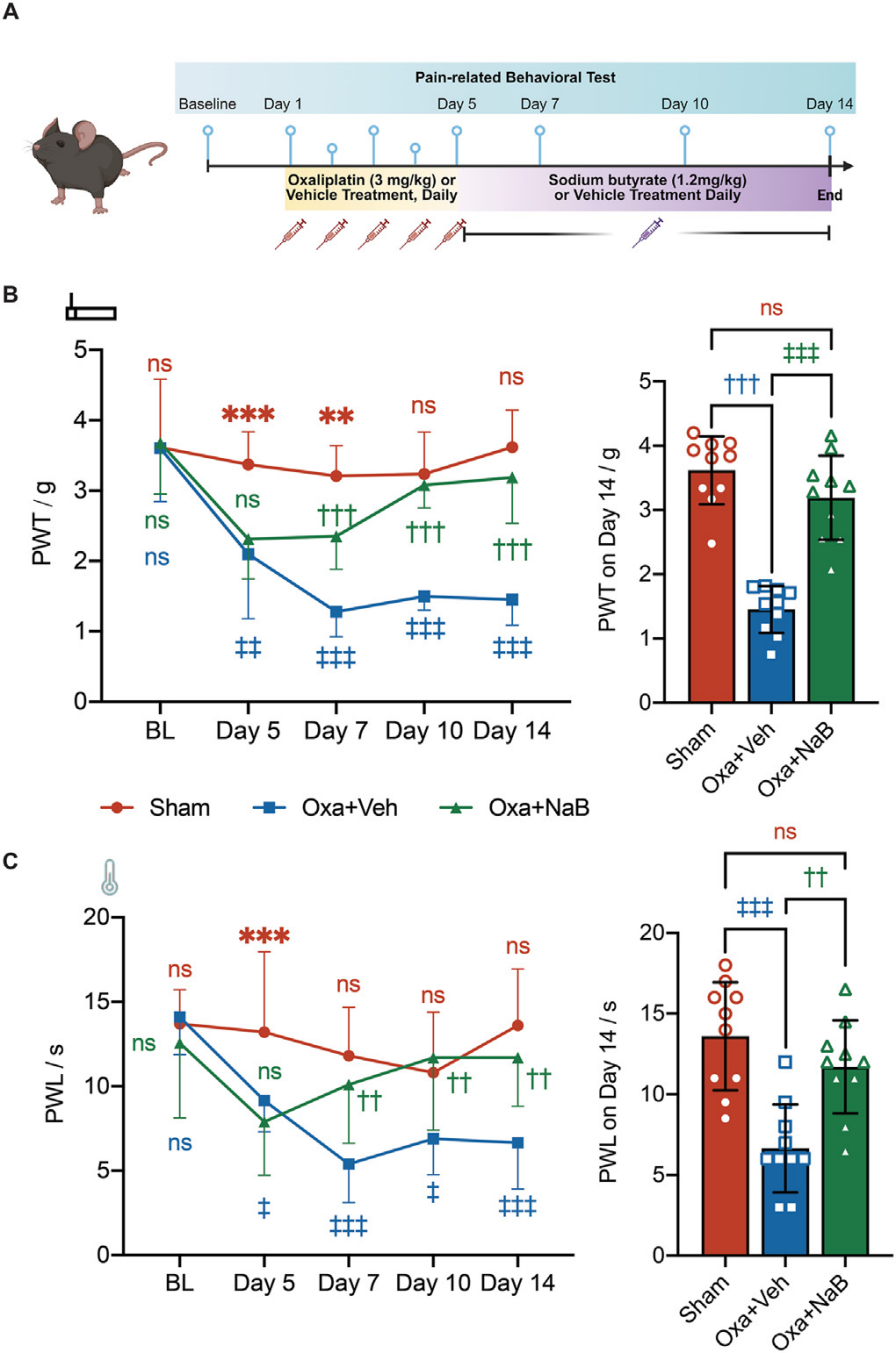
**Sodium butyrate ameliorated oxaliplatin-mediated CIPN-related pain-like behavior in mice.** **A**, An illustration of the experimental timeframe for pain-related behavioral tests. Following consecutive injection of 3 ​mg/kg oxaliplatin (Oxa) or vehicle (Veh) for 5 days, male and female mice were treated daily with 1.2 ​mg/kg sodium butyrate (NaB) or vehicle (Veh) until the end of the experiment. Pain-related behaviors were tested with electronic von Frey and acetone test on baseline (BL), day 5, 7, 10 and 14 after the first injection of oxaliplatin. **B**, ***Left***, paw withdrawal thresholds (PWT) against von Frey of male and female mice. ***Right***, PWT of male and female mice on day 14 post-first oxaliplatin injection. **C**, ***Left***, paw withdrawal latencies (PWL) to acetone challenge of male and female mice. ***Right***, PWL of male and female mice on day 14 post-first oxaliplatin injection. Two-way ANOVA followed by Tukey's multiple comparisons test, *n* ​= ​10 per group. ns, non-significant. Sham (Veh+Veh) vs Oxa+Veh, ‡, *p* ​< ​0.05, ‡‡, *p* ​< ​0.01, ‡‡‡, *p* ​< ​0.001. Sham vs Oxa+NaB, ∗∗*p* ​< ​0.01, ∗∗∗*p* ​< ​0.001. Oxa+Veh vs Oxa+NaB, ††*p* ​< ​0.01, †††*p* ​< ​0.001.

Sodium butyrate (Cayman Chemical, Ann Arbor, MI, USA) dissolved in sterile normal saline was injected intraperitoneally at a concentration of 1.2 ​mg/kg every day following oxaliplatin injection until the end of the experiment. Control animals received sterile normal saline prepared in our laboratory as vehicle injection (Fig. 3A).

### Pain-related behavioral tests

All mice were acclimated to the testing environment 3–5 days prior to tests. Researchers were blinded to the animal grouping conditions for the pain-related behavioral tests. All behavioral tests were conducted between 9:00–16:30 ​h.

To measure mechanical hypersensitivity, electronic Von Frey system was used [31]. Each mouse was put in an individual Plexiglas chamber on an elevated mesh for about an hour before the test. The semi-flexible tip connected to an electronic von Frey Anesthesiometer (IITC Inc. Life Science Instruments, Woodland Hills, CA, USA) was applied to the plantar surface of the left hind paw of each mouse. A mechanical stimulus-related withdrawal of the tested paw was considered a positive withdrawal response. The pressure threshold value (in g) to trigger a positive paw withdrawal response was recorded. The average threshold across 5 test trials per mouse with an inter-stimulation period of 5 ​min were reported as paw withdrawal threshold (PWT).

Acetone evaporation test was adopted to test for cold hypersensitivity [31,32]. After habituation in an individual Plexiglas chamber on an elevated mesh for about 1 ​h, 25 ​μL of acetone was applied onto the plantar surface of the left hind paw of each mouse. The latencies (in s) to a positive paw withdrawal, defined as flinching, licking or biting of the stimulated paw over a 60-s period was recorded. The average latency across 5 test trials per mouse with an inter-stimulation period of 5 ​min were reported as paw withdrawal latencies (PWL).

### Primary dorsal root ganglion neuronal culture

The vertebral column was dissected from terminally anaesthetized mice (5–6 weeks). A sagittal cut was made to the vertebral column to expose the neuroforamen and the DRGs. L3-L6 DRGs were collected with fine forceps and put into Dulbecco's modified Eagle's medium: Nutrient Mixture F-12 (DMEM/F12, Gibco, Waltham, MA, USA) on ice. Fibers were removed carefully under a dissection microscope. The DRGs were incubated in 2 ​mg/mL collagenase Type 1A (Gibco, Waltham, MA, USA) and 5 ​mg/mL dispase (Gibco, Waltham, MA, USA) in DMEM/F12 at 37 ​°C for 1 ​h. The digestive enzyme solution was discarded and the DRGs were gently rinsed with DMEM/F12 once before the neurons were resuspended in fresh DMEM/F12 supplemented with 1 ​× ​B27 (Gibco, Waltham, MA, USA) and 1 ​× ​Penicillin-Streptomycin (Gibco, Waltham, MA, USA). The DRG solution was filtered with a 70 ​μm cell strainer (SPL, Pochon, Gyeonggi-do, Korea) and the neurons were allowed to sink at 37 ​°C for 30 ​min. The neurons at the bottom were collected to plate onto 24 well plates or confocal dishes pre-coated with poly-L-lysine (Merck Millipore, Burlington, MA, USA) and laminin (Sigma-Aldrich, St. Louis, MO, USA) before subsequent experiment.

### Real time-quantitative polymerase chain reaction (rt-qPCR)

Total RNA of L3-L6 DRG tissues of mice post-vehicle or oxaliplatin injection was extracted with RNAiso Plus (Takara Bio Inc., Kusatsu, Shiga, Japan) and reverse transcribed into cDNA with PrimeScript RT Master Mix (Takara Bio Inc., Kusatsu, Shiga, Japan). The gene expression levels of pro-inflammatory cytokines (*Il-1β*, *Il-6* and *Tnf-α*), HDAC subtypes (*Hdac1*, *Hdac2*, *Hdac3*, *Hdac4*, *Hdac5, Hdac6*, *Hdac7*, *Hdac8*, and *Hdac9*), pain-related potassium channels (*Kcna1*, *Kcnd2*, *Kcnj3*, *Kcnj6*, *Kcnj9*, *Kcnk2*, *Kcnk3*, *Kcnk4*, *Kcnk9*, *Kcnk10*, *Kcnk18*, *Kcnq2* and *Kcnt1*) and the housekeeping genes (*β-tubulin* and *GAPDH*) was quantified with TB Green Premix Ex Taq II (Takara Bio Inc., Kusatsu, Shiga, Japan) according to manufacturer's instruction with primers in Table 1. The relative expression of each interested gene was normalized to *β-tubulin* and compared with the corresponding control using the delta-delta Ct method.

**Table 1:** List of primers used in rt-qPCR.

Gene	Upper (5′ to 3′)	Lower (5′ to 3′)
*Hdac1*	GCCAAAGGGGTCAAAGAAG	TGAGGAACTTGGGGAGAAGA
*Hdac2*	TCCGGTGTTTGATGGACTCT	CCCCAGCAACTGAACCAC
*Hdac3*	CCCGAGGAGAACTACAGCAG	TCCTTGTCGTTGTCATGGTC
*Hdac4*	GCCATCTGTGATGCTTCTGA	GAGTGGACAGCATTGGCATT
*Hdac5*	TCAACTCCGTAGCCATCACA	GAATATCCCAGTCCACGATGA
*Hdac6*	ACTCATGGGGGCTGAGATTC	TGAACATGCAATACCCATCC
*Hdac7*	GGGTGCACAGGAAATACTGG	GGGTAGCCAGGAGTCTGGA
*Hdac8*	GCCTATGCCCTGCATAAACA	AGGCATCAGTGTGGAAGGTG
*Hdac9*	TTCCTGGAGAAGCAGAAACA	GAGGCTGCTCTGTCTTCCAT
*Kcna1*	AGTGCAGCTTATCGCCATTT	GTGCAAGCAACCTGGAGAC
*Kcnd2*	TGTCGAACTTCAGTCGGATCT	CTTTCTTCTGTGCCCTTCGT
*Kcnj3*	TTGTGGAAACCACAGGAATG	CAAAGCACTTCGTCCTCTGT
*Kcnj5*	ATGTCTCGTGCTCAACTGGA	CAAGTCATGCCTGTTGCTTC
*Kcnj6*	TCCAGCAAACTGAACCAACA	TCACCCATTCCTCTCCGTC
*Kcnj9*	CCTGCTGCTGGCTACTCTTC	GAGTCGGTTCCTAGGCTTTCA
*Kcnk2*	TCTGAGCATGATTGGGGACT	CCACCTCTTCCTTCGTCTTC
*Kcnk3*	AGGCAAGGTGTTCTGCATGT	GCAGGTACCTCACGAAGGTG
*Kcnk9*	ACCGATGAGGAGCTACTGGA	CACAGGGTTCTGCCGTATTT
*Kcnk10*	TGATTCAGCATGCACTCGAT	TGCTGTTGGAAGAGTTTCCT
*Kcnk18*	CAGGACTGGTCCTTCCTGAG	ATGTGGCCATAACCCACTGT
*Kcnq2*	CACGCCTACGTGTTCCTTTT	ATGGTGGAAAACACAGAAAGC
*Kcnt1*	AAGACTGGAACCCAACGACA	TTCCGACTCTGGGAGCTG
*kcnk4*	GTGACTCTCACCACTGTAGGCT	TGGTGAGCACTGAGGCGAAGTA
*β-tubulin*	CTGGACCGCATCTCTGTGTACT	GCCAAAAGGACCTGAGCGAACA
*GAPDH*	GTATGACTCCACTCACGGCAA	GGTCTCGCTCCTGGAAGATG
*Tnf*	AAATTCGAGTGACAAGCCTGTAG	GAGAACCTGGGAGTAGACAAGGT
*Il6*	AAGCCAGAGTCCTTCAGAGAGATA	ATTTCAAGATGAATTGGATGGTCT
*Il1b*	TGGACCTTCCAGGATGAGGACA	GTTCATCTCGGAGCCTGTAGTG
*Ffar3*	GGGCAGCAGAGTGCCAGTTGT	AACGTTCGATGCTCACCGCCG
*Ffar2*	AGGTAGCGTTCCATGCTGAT	GCACTGGACCAGAGGAGAAC
*Hcar2*	CCGGACGGCGGCCATCATTT	ATGCGCACAGCCACACTGGG

### Immunofluorescence (IF)

For visualizing proteins in DRG tissues, terminally anaesthetized mice were myocardially perfused with normal saline (0.9 ​% NaCl) followed by 4 ​% paraformaldehyde (PFA). DRGs were isolated and post-fixed in 4 ​% PFA overnight, followed by cryopreservation with 30 ​% sucrose solution in 0.1 ​M phosphate-buffered saline (PBS), pH 7.4 for 72 ​h. DRG tissues were then sectioned and mounted onto slides before IF staining. For visualizing proteins in isolated DRG cultures, primary cells prepared in confocal dishes were fixed in 4 ​% PFA for 10 ​min before IF staining.

The samples were washed 3 times with PBS for 6 ​min before incubating with blocking buffer [10 ​% normal goat serum (Gioco, Waltham, MA, USA), 0.03 ​% Triton X-100 (Sigma-Aldrich, St. Louis, MO, USA) in PBS] for 1 ​h. Blocked samples were then incubated with primary antibodies against HDAC1 (#YT2145, ImmunoWay Biotechnology, Planto, TX, USA), AcH3K9 (#YK006, Immunoway, Plano, TX, USA), AcH3K14 (#07–353, Merck Millipore, Burlington, MA, USA), Kcnk18 (#APC-122, Alomone Labs, Jerusalem, Israel), NeuN (#ABN90, Merck Millipore, Burlington, MA, USA), or β3-Tubulin (#YM3165, ImmunoWay Biotechnology, Planto, TX, USA) in fresh blocking buffer at 4 ​°C overnight. Samples were then washed 3 times with PBS for 6 ​min before incubating with secondary Alexa Fluor antibodies purchased from Life Technolgies, Carlsbad, CA, USA: Alexa Fluor 633 goat anti-guinea pig IgG (H+L) (# A21105), Alexa Fluor 555 donkey anti-mouse IgG (H+L) (#A31570), Alexa Fluor 555 goat anti-rabbit IgG (H+L) (#A21428 Life Technologies), Alexa Fluor 488 goat anti-rabbit IgG (H+L) (A11001), Alexa Fluor 488 goat anti-mouse IgG (H+L) (A11073), or Alexa Fluor 633 goat anti-mouse IgG (H+L), highly cross adsorbed (A21052) Cell Signaling Technology, Inc. Danvers, MA, USA) for 2 ​h. The samples were then washed 3 times with PBS, counter stained with DAPI (Merck Millipore, Burlington, MA, USA) for 10 ​min, washed 3 times again and mounted with VECTASHIELD Antifade Mounting Medium (Vector Laboratories, Burlingame, CA, USA). Fluorescence images were captured on a Leica SP8 confocal microscopy Platform (Leica, Wetzlar, Germany).

### Western blotting

L3-6 DRG tissues were freshly isolated from mice and lysed in radioimmunoprecipitation assay (RIPA) lysis buffer. Protein concentration was determined with Pierce BCA Protein Assay Kit (Thermo Scientific, Waltham, MA, USA). Lysed proteins were heated in 4X Protein SDS-PAGE loading buffer (Takara Bio Inc., Kusatsu, Shiga, Japan) at 100 ​°C for 10 ​min. Proteins were separated on 10–15 ​% SDS-PAGE and transferred onto PVDF membranes (Merck Millipore, Burlington, MA, USA). Membranes were incubated in blocking buffer for 1 ​h before primary antibodies against HDAC1 (#YT2145, ImmunoWay Biotechnology, Plano, TX, USA), AcH3K9 (#YK006, Immunoway, Plano, TX, USA), AcH3K14 (#07–353, Merck Millipore, Burlington, MA, USA), b-actin (#4697, Cell Signaling technology, Danvers, MA, USA) or Tubulin (#2146S, Cell Signaling technology, Danvers, MA, USA) at 4 ​°C overnight. The membranes were then washed with Tris-buffered saline with Tween 20 (TBST) 3 times before incubated with Anti-rabbit IgG, HRP-linked secondary antibodies (#7074, Cell Signaling Technology, Inc. Danvers, MA, USA) or Goat anti-Mouse IgG (H+L) Secondary Antibody, HRP (#331430, Invitrogen, Waltham, MA, USA). The membranes were washed with TBST for 3 times before detection with Pierce ECL Western Blotting Substrate (Thermo Scientific, Waltham, MA, USA) using Protect Optimax X-ray Film Processor (Protec, Oberstenfeld, Germany). The X-ray films were imaged with a HP Scanjet 4890 (HP, Palo Alto, CA, USA). Semi-quantification of the protein bands was done with ImageJ.

### Current-clamp and voltage recordings

Animals were deeply anaesthetized with urethane (1.5 ​g/kg, i.p.) for isolation of DRG tissues. DRGs were enzymatically dissociated as the preparation of primary DRG neuronal culture and plated onto coverslips. Patch pipettes were pulled from borosilicate capillaries (World Precision Instruments, Inc.) using a P-97 micropipette puller (Sutter Instrument Co.). Whole cell patch clamp recordings were performed at room temperature with an Axon patch 700B amplifier (resistance of patch pipettes, 10 ​MΩ) with a Digidata 1440A digitizer (Axon Instruments, holding potential, −60 ​mV) within 1–2 ​h after plating the cells into culture dish. The pipette solution contained the following (in mM): 130 ​K-gluconate, 7 KCl, 2 NaCl, 0.4 EGTA, 1 MgCl2, 4 ATP-Mg, 0.3 GTP-Na, 10 HEPES, 10 Tris-phosphocreatine, 10 ​U/ml creatine phosphokinase, pH 7.3 with KOH, and 290 mOsmol/kg H2O. Recordings were obtained from small DRG neurons (<25 ​μm) perfused with Tyrode's solution (∼0.5 ​mL/min) containing the following (in mM): 130 NaCl, 2 KCl, 2 CaCl2, 2 MgCl2, 25 HEPES, 30 Glucose, pH 7.3 with NaOH, and 310 mOsmol/kg H2O. For current clamp recordings, action potentials were triggered by current injection steps from 0 to 130 ​pA with 10 ​pA increments over 450 ​ms. Background K^+^ currents and TRESK-mediated background currents were measured via bath-applied 30 ​μM lamotrigine (Sigma-Aldrich, St Louis, MO, USA) while evoking whole cell currents in neurons perfused with Tyrode solution contained 1 ​μM tetrodotoxin (TTX) as described before [[33], [34], [35], [36], [37]]. Neurons were held at −60 ​mV and depolarized to −25 ​mV for 150 ​ms before the potential was ramped to −135 ​mV at a rate of 0.37 ​mV/ms every 10 ​s. The outward currents at the end of the −25 ​mV depolarizing step were recorded as the total outward current. Data were analyzed using the pClamp10 (Axon Instruments) software.

### Statistical analyses

Based on differences in preliminary pain behavioral test for mechanical allodynia between vehicle and oxaliplatin groups (2.62 ​± ​0.72 ​g vs 1.41 ​± ​0.59 ​g), a sample size of n ​= ​6 animals per group is sufficient to attain statistical significance of p ​< ​0.05 with a 90 ​% power. In the behavioral test involving butyrate treatment, 5 female and 5 male mice were included. All mice were randomly assigned into experimental groups.

All statistical tests were done using Prism 9 software. For all parametric tests, the data was tested for normal distribution and equal variance before followed by unpaired *t*-test. Paired *t*-test was used when specified. For comparing gene or protein expression on more than 2 time points, or percentage of lamotrigine-sensitive persistent K^+^ currents inhibition, one-way analysis of variance (ANOVA) with Tukey's multiple comparison test was used. Differences in paw withdrawal thresholds or latencies, or number of evoked action potential, were tested with two-way repeated measures ANOVA with Tukey's multiple comparison test. A two-tailed p-value less than 0.05 was considered statistically significant. All data were represented as mean ​± ​S.D. unless stated otherwise.

## Results

### Oxaliplatin induced pain-like behavior in mice

We established an oxaliplatin-mediated CIPN model by exposing mice to a cumulative dose of 15 ​mg/kg oxaliplatin by intraperitoneal injection (i.p.) at 3 ​mg/kg daily for 5 consecutive days (Fig. 1A). Oxaliplatin treatment impaired the increase of body weight by 6.46 ​% (20.50 ​± ​0.84 ​g vs 21.92 ​± ​0.49 ​g, *p* ​= ​0.01) on day 14 and 5.62 ​% on day 21 (22.25 ​± ​0.40 ​g vs 21.00 ​± ​0.26 ​g, *p* ​= ​0.04) post-injection when compared to vehicle (Fig. 1B). Immediately following the oxaliplatin injection course, marked mechanical allodynia was evoked in oxaliplatin group from day 5 with 46.4 ​% decrease in paw withdrawal thresholds (1.39 ​± ​0.23 ​g vs 2.59 ​± ​0.33 ​g) compared with the vehicle group (Fig. 1C). The oxaliplatin-induced mechanical allodynia lasted for at least 21 days with a 40.5 ​% decrease in paw withdrawal thresholds in the oxaliplatin group (1.62 ​± ​0.33 ​g vs 2.72 ​± ​0.57 ​g), compared with the vehicle group (Fig. 1C). The deficiency in body weight gain and development of mechanical allodynia were consistent with previous studies and supported the presence of oxaliplatin-mediated CIPN. In L3-6 DRGs, *Il-1β*, *Il-6* and *Tnf-α* were upregulated by 2.8-fold (*p*-value ​= ​0.0021), 2.0-fold (*p*-value ​= ​0.0015) and 3.2-fold (*p*-value ​= ​0.0006), respectively on day 14 after injection (Fig. 1D–F). Taken together, oxaliplatin-induced neuropathic pain model was successfully established in the present study.

### Upregulation of HDAC1 activity in DRGs after OIPN

Previous studies revealed that HDACs are involved in nociceptive hypersensitivity involved in the generation of neuropathic or inflammatory pain [16,38,39]. Intrathecal delivery of MS-275 and MGCD0103, two different class I HDACIs, reduced hind paw hypersensitivity by about 40 ​% in rats several rat models of nerve injury- or antiviral drug-induced neuropathic pain [16]. In the present study, to identify the members in HDACs family that were dysregulated in DRGs after oxaliplatin-mediated CIPN, we extracted total RNA from L3-6 DRGs of mice underwent vehicle or oxaliplatin treatment and examined the expression levels of HDAC members on day 14 by qualitative PCR. Among HDAC subtypes 1–9, only *Hdac1* expression was significantly induced by oxaliplatin injection (1.8 ​± ​0.2-fold compared with vehicle, *p* ​= ​0.005, Fig. 2A). The enhanced expression was transient and returned to baseline on day 21 after the first dose of oxaliplatin injection (Fig. 2B). We determined if oxaliplatin regulated the protein level of HDAC1 in DRG tissues by immunohistochemistry assay. The mean fluorescence intensity increased from 10.5 ​± ​3.6 to 17.5 ​± ​4.4 units in DAPI-positive regions in *β*III tubulin-positive neuronal components of DRG tissues (*p* ​< ​0.001, vehicle compared with oxaliplatin treatment, Fig. 2C). Our Western blot assay further confirmed HDAC1 transiently increased at protein level after oxaliplatin injection (day 7: 2.6 ​± ​0.7-fold, *p* ​= ​0.03; day 9: 4.1 ​± ​0.6-fold, *p* ​< ​0.001, compared with vehicle, Fig. 2D). The upregulation of HDAC1 in DRG tissues contributed to the development of pain hypersensitivity after oxaliplatin injection.

### Sodium butyrate ameliorated mechanical and cold hypersensitivity after oxaliplatin-induced neuropathy

**Fig. S1:**
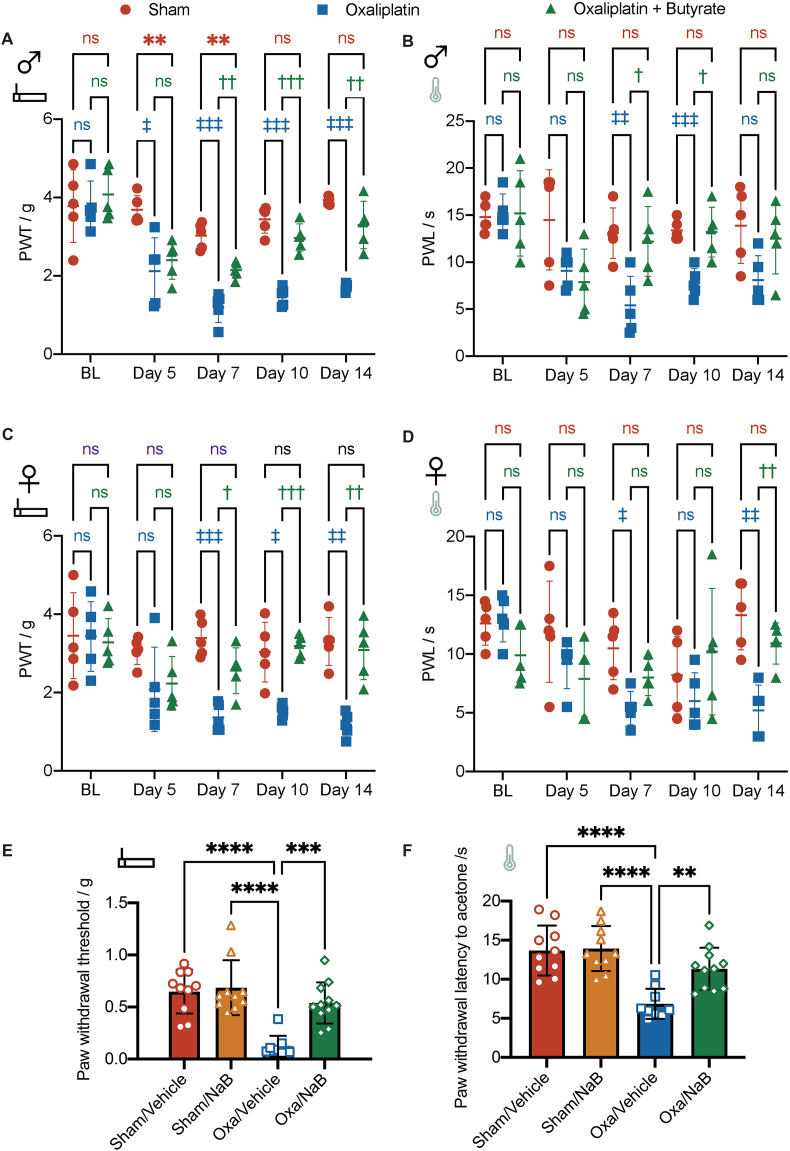
**Sodium butyrate ameliorated oxaliplatin-mediated CIPN-related pain-like behavior in both male and female mice.** Raw data points of male and female in Fig. 3. **A**, paw withdrawal thresholds (PWT) against von Frey of male mice. **B**, paw withdrawal latencies (PWL) to acetone challenge of male mice. **C**, PWT against von Frey of female mice. **D**, PWL to acetone challenge of female mice. Two-way ANOVA followed by Tukey's multiple comparisons test, *n* ​= ​5 per group for each sex. ns, non-significant. Sham (Veh+Veh) vs Oxa+Veh, ‡, *p* ​< ​0.05, ‡‡, *p* ​< ​0.01, ‡‡‡, *p* ​< ​0.001. Sham vs Oxa+NaB, ∗∗*p* ​< ​0.01. Oxa+Veh vs Oxa+NaB, ††*p* ​< ​0.01, †††*p* ​< ​0.001. **E**, PWT against von Frey of male mice 21 days post first oxaliplatin dose. **F**, PWL to acetone challenge of male mice 21 days post first oxaliplatin dose. One-way ANOVA followed by Tukey's multiple comparisons test, *n* ​= ​9–11 male mice per group. ∗∗*p* ​< ​0.01. ∗∗∗*p* ​< ​0.001. ∗∗∗∗*p* ​< ​0.0001.

Administration of sodium butyrate decreased the visceral perception of pain compared with placebo in healthy volunteers [29]. In preclinical studies, NaB significantly attenuated chronic constriction injury-induced pain-like behavior in rats [24]. To test whether NaB attenuates CIPN-induced pain hypersensitivity, we administered NaB by daily intraperitoneal injection to both male and female mice underwent vehicle or oxaliplatin injection (Fig. 3A and Supplementary Fig. 1A–D). The oxaliplatin-induced mechanical allodynia was reversed by NaB administration from day 7 after the first dose of oxaliplatin and lasted for at least 14 days (Oxa+NaB vs Oxa+Veh. Day 7: 2.4 ​± ​0.5 ​s vs 1.3 ​± ​0.3 ​s, *p* ​< ​0.001; day 10: 3.1 ​± ​0.3 ​s vs 1.5 ​± ​0.2 ​s, *p* ​< ​0.001; day 14: 3.2 ​± ​0.7 ​s vs1.5 ​± ​0.4 ​s, *p* ​< ​0.001, Fig. 3B). As oxaliplatin-induced neuropathy also manifested cold hypersensitivity in nearly all patients under the oxaliplatin chemotherapy [40], we also investigated if NaB reversed oxaliplatin-induced cold hypersensitivity. The paw withdrawal latencies to acetone spray were significantly reduced in the mice model of oxaliplatin-mediated CIPN from day 5 to day 14 when compared to sham group (Oxa+Veh vs Sham. Day 5: 9.15 ​± ​1.8 ​s vs 13.2 ​± ​4.8 ​s, *p* ​< ​0.05; Day 7: 5.4 ​± ​2.3 ​s vs 11.8 ​± ​2.9 ​s, *p* ​< ​0.001; day 10: 6.9 ​± ​2.1 ​s vs 10.8 ​± ​3.6 ​s, *p* ​< ​0.05; day 14: 6.7 ​± ​2.7 ​s vs 13.6 ​± ​3.4 ​s, *p* ​< ​0.001, Fig. 3C). Administration of NaB reversed the oxaliplatin-induced cold hyperalgesia-like behavior when compared to vehicle group (Oxa+NaB vs Oxa+Veh. Day 7: 10.1 ​± ​3.5 ​s vs 5.4 ​± ​2.3 ​s, *p* ​< ​0.01; day 10: 11.7 ​± ​4.3 ​s vs 6.9 ​± ​2.1 ​s, *p* ​< ​0.01; day 14: 11.7 ​± ​2.9 ​s VS 6.7 ​± ​2.7 ​s, *p* ​< ​0.01, Fig. 3C). The paw withdrawal latencies to acetone spray of the Oxa+NaB mice was comparable to the Sham group (p ​> ​0.05 for day 7, 10 and 14 respectively, Fig. 3C). The beneficial effects of butyrate on pain hyper-sensitivity persisted by day 21 (Supplementary Fig. 1E and F). Collectively, these data suggested that daily administration of NaB provided analgesic-like effects in the mice model of oxaliplatin-mediated CIPN, regardless of sex.

### Sodium butyrate increased histone acetylation in DRG neurons

**Fig. 4:**
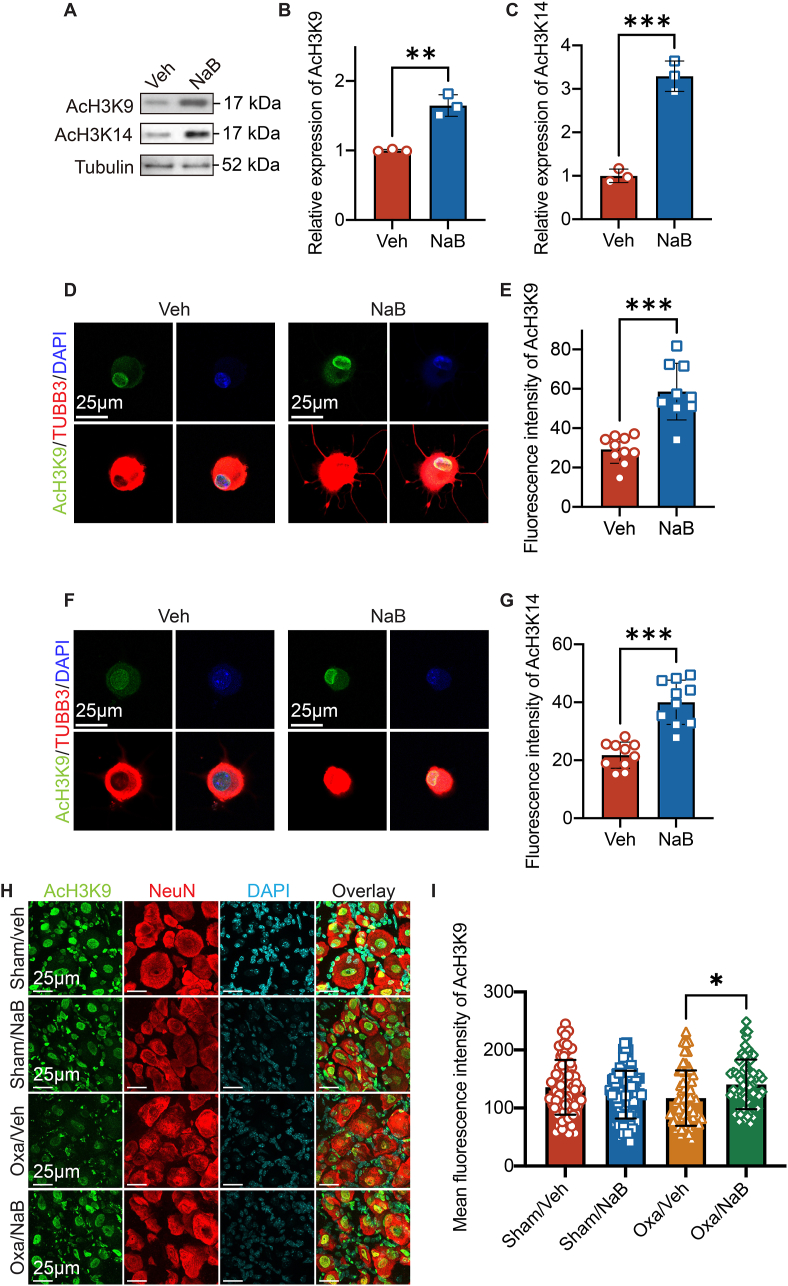
**Sodium butyrate increased global histone acetylation in DRG neurons.** **A**, Representative Western blot of AcH3K9 or AcH3k14 in isolated L3-L6 DRG tissues incubate with 2 ​μM of sodium butyrate (NaB) or vehicle (Veh) for 6 ​h. **B and C**, Densitometry quantification of AcH3K9 or AcH3K14 protein content normalized with tubulin. Unpaired *t*-test, *n* ​= ​3 DRG neuronal cultures per group. ns, non-significant, ∗∗*p* ​< ​0.01, ∗∗∗*p* ​< ​0.001. **D and F**, Representative immunofluorescence of AcH3K9 or AcH3K14 (Green), *β*III Tubulin (TUBB3, Red) and DAPI (Blue) in L3-L6 DRG neuronal culture incubated with NaB or Veh for 6 ​h. **E and G**, Quantification of mean fluorescence intensity of AcH3K9 or AcH3K14 in nuclei of DRG neurons. Unpaired *t*-test, *n* ​= ​10 DRG neurons from 3 independent male mice per group. ns, non-significant, ∗∗∗*p* ​< ​0.001. **H**, Representative immunofluorescence of AcH3K9 (Green), NeuN (Red) and DAPI (cyan) in L3-L6 DRG tissues collected at day 21 post first dose of oxaliplatin. Male mice were treated with sham or oxaliplatin injection followed by sodium butyrate. **I**. Quantification of mean fluorescence intensity of AcH3K9 in nucleus of NeuN-positive cells. One-way ANOVA followed by Tukey's multiple comparisons test, *n* ​= ​at least 60 ​cells from 3 to 5 male mice per group. ns, non-significant, ∗*p* ​< ​0.05.

**Fig. S2:**
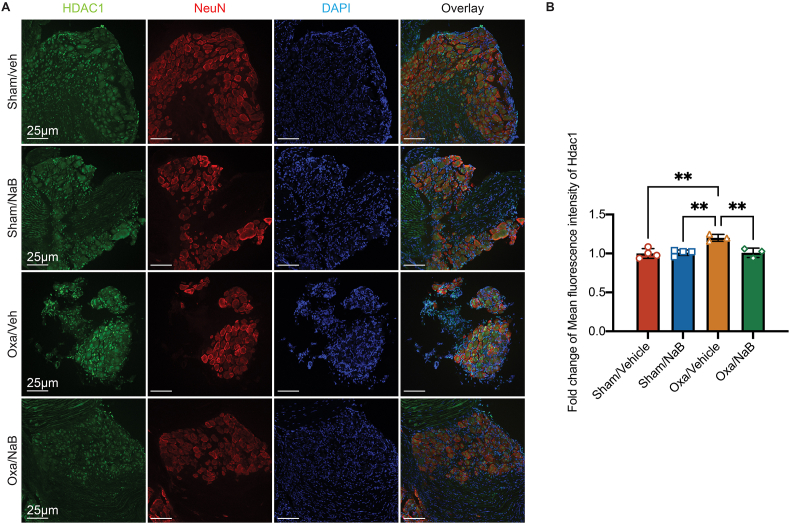
**Oxaliplatin-induced enhanced expression of HDAC1 in DRG tissues was demolished by sodium butyrate.** **A**, Representative immunofluorescence of HDAC1 (Green), NeuN (Red) and DAPI (blue) in L3-L6 DRG tissues collected at day 21 post first dose of oxaliplatin. Male mice were treated with sham or oxaliplatin injection followed by sodium butyrate. **I**. Quantification of mean fluorescence intensity of HDAC1 in nucleus of NeuN-positive cells. One-way ANOVA followed by Tukey's multiple comparisons test, *n* ​= ​at least 60 ​cells from 3 to 5 male mice per group. ns, non-significant, ∗∗*p* ​< ​0.01.

We then explored the underlying mechanism of NaB-induced analgesic actions on oxaliplatin-mediated CIPN-induced pain hypersensitivity. Our data suggested that the development of pain hypersensitivity was accompanied by upregulation of HDAC1 in DRG tissues. As NaB is a known class I and IIa HDACI [12,30], we determined if NaB increased global histone acetylation of Lysine residues in primary mice DRG neuronal cultures. Compared with vehicle, incubation of isolated L3-6 DRGs with 2 ​μM of NaB for 6 ​h induced a significant increase in acetylation of histone H3 at lysine 9 (AcH3K9, 1.5 ​± ​0.2-fold compared to vehicle group, Fig. 4A and B) and at lysine 14 (AcH3K14, 3.3 ​± ​0.4-fold compared to vehicle group, Fig. 4A and C) as detected by the Western blot assay. Double staining with anti-βIII-tubulin antibodies confirmed that the NaB-induced increase in global histone acetylation at H3K9 (2.0 ​± ​0.5-fold compared to vehicle group, Fig. 4D and E) and H3K14 (1.8 ​± ​0.4-fold compared to vehicle group, Fig. 4F and G) was found in the nucleus of DRG neurons. In animal DRG tissues, HDAC1 expression in neuronal nucleus was increased in oxaliplatin group compared with sham animals, while this was reversed by sodium butyrate (Supplementary Fig. 2). AcH3K9 levels were significantly increased in mice treated with oxaliplatin and sodium butyrate when compared with mice treated with oxaliplatin alone (Fig. 4H and I). These data suggested that NaB increased *in vivo* global histone acetylation of Lysine residues in DRG tissues challenged by oxaliplatin.

### Sodium butyrate induced transcriptional remodeling of potassium channel expression in DRGs

**Fig. 5:**
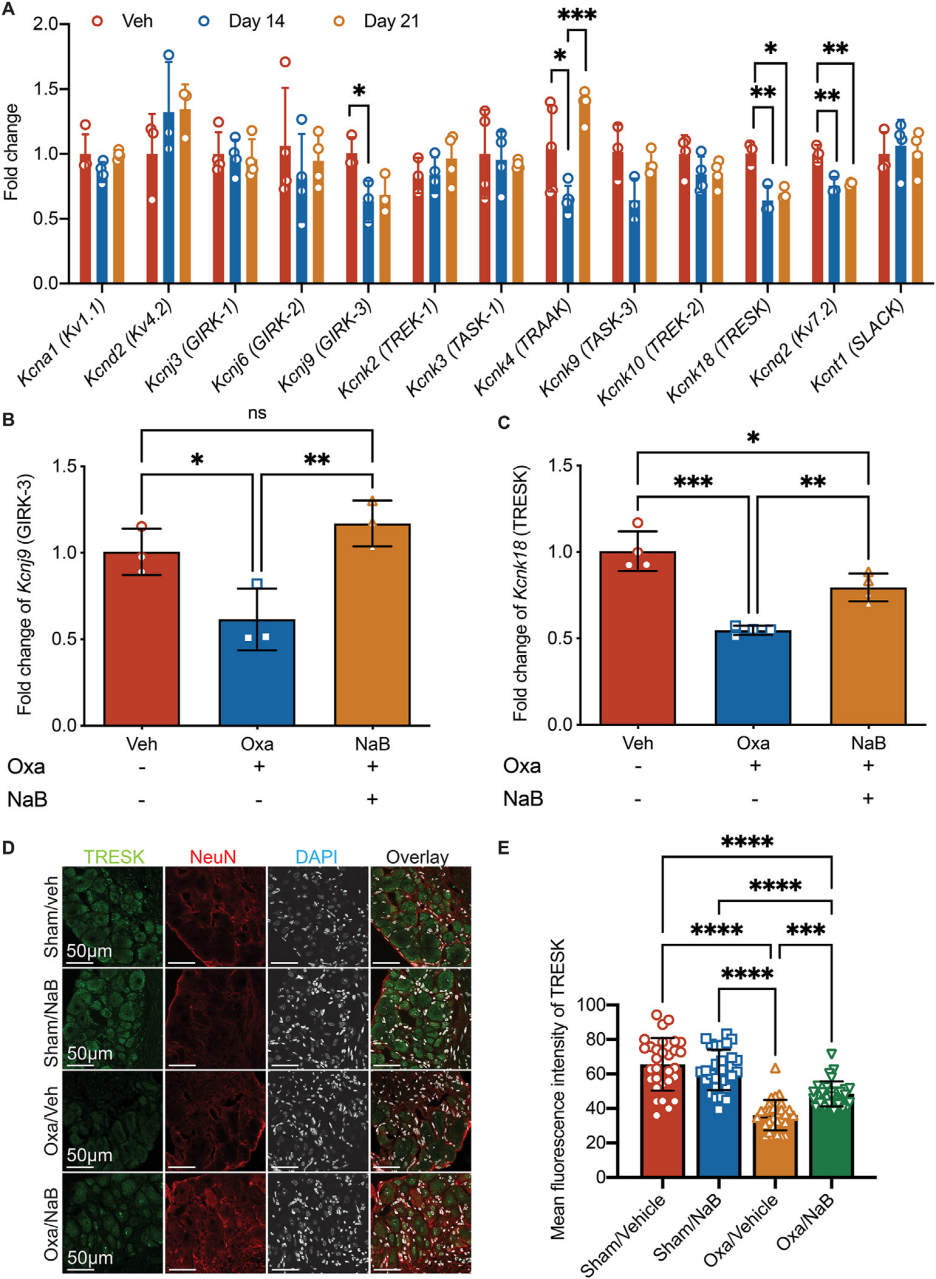
**Sodium butyrate reversed oxaliplatin-induced repression of *Kcnj9* and *Kcnk18* expression.** **A**, Expression levels of potassium channels encoding genes selected from the Pain Gene Database in DRG tissues of mice receiving oxaliplatin (Oxa) or vehicle (Veh) on day 14 and 21 post-injection. One-way ANOVA followed by Tukey's multiple comparisons test comparing Veh and Oxa group per gene, *n* ​= ​3–4 per group. *p*-values not shown were of no significant difference, ∗*p* ​< ​0.05, ∗∗*p* ​< ​0.01. **B and C**, Expression levels of *Kcnj9* or *Kcnk18* of sham mice followed by vehicle treatment (Veh), male animals received oxaliplatin followed by vehicle treatment (Oxa) or male animals received oxaliplatin followed by sodium butyrate treatment (NaB) on day 14. One-way ANOVA followed by Tukey's multiple comparisons test, *n* ​= ​3–4 per group. ns, non-significant, ∗*p* ​< ​0.05, ∗∗*p* ​< ​0.01, ∗∗∗*p* ​< ​0.001. **D**, Representative immunofluorescence of TRESK (Green), NeuN (Red) and DAPI (white) in L3-L6 DRG tissues collected at day 21 post first dose of oxaliplatin. Male mice were treated with sham or oxaliplatin injection followed by sodium butyrate. **E**. Quantification of mean fluorescence intensity of TRESK in NeuN-positive cells. One-way ANOVA followed by Tukey's multiple comparisons test, *n* ​= ​at least 30 ​cells from 3 to 5 male mice per group. ns, non-significant, ∗*p* ​< ​0.05.

It has been reported that a majority of DRG-expressing potassium (K^+^) channels was reduced, following peripheral nerve injuries, diabetic neuropathy or bone cancer pain, while some of them were proved critical for neuropathic pain hypersensitivity [41]. In our CIPN model, mRNA levels of 13 DRG-expressing K^+^ channel subunits relevant to pain were analyzed by real-time qPCR. On day 14 post-first oxaliplatin injection, *Kcnj9*, *Kcnk18, Kcnk4* and *Kcnq2* were significantly down-regulated in the L4-L5 DRG tissues of oxaliplatin-treated compared with vehicle-treated mice (*Kcnj9*: 0.6 ​± ​0.2-fold, *p* ​< ​0.05; *Kcnk18*: 0.6 ​± ​0.1-fold, *p* ​< ​0.01; *Kcnk4*: 0.65 ​± ​0.1-fold, *p* ​< ​0.05, *Kcnq2*: 0.8 ​± ​0.1-fold, *p* ​< ​0.01, Fig. 5A). The reduction of *Kcnk18* and *Kcnq2* sustained until at least day 21 (*Kcnk18*: 0.7 ​± ​0.1-fold, *p* ​< ​0.05; *Kcnq2*: 0.8 ​± ​0.1-fold, *p* ​< ​0.01, Fig. 5A). Treatment with NaB completely abolished the reduction of *Kcnj9* and *Kcnk18* in DRG tissues of mice receiving oxaliplatin injections, compared to the Oxa+Veh group (Fig. 5B and C), while NaB exerted no effect on *Kcnq2* expression (Data not shown). In animal DRG tissues, *Kcnk18* (TRESK) expression in neuronal nucleus was decreased in oxaliplatin group compared with sham animals, while this was reversed by sodium butyrate (Fig. 5D and E). Treatment with MS-275, a class one HDAC inhibitor with pain relieving properties [15], mimicked sodium butyrate functions and resulted in restored TRESK expressions in mice receiving oxaliplatin, further suggesting oxaliplatin-induced TRESK channel downregulation was related to HDAC inhibition (Supplementary Fig. 4).

### Sodium butyrate repressed TRESK-induced hyperexcitability of DRG neurons in OPIN

**Fig. 6:**
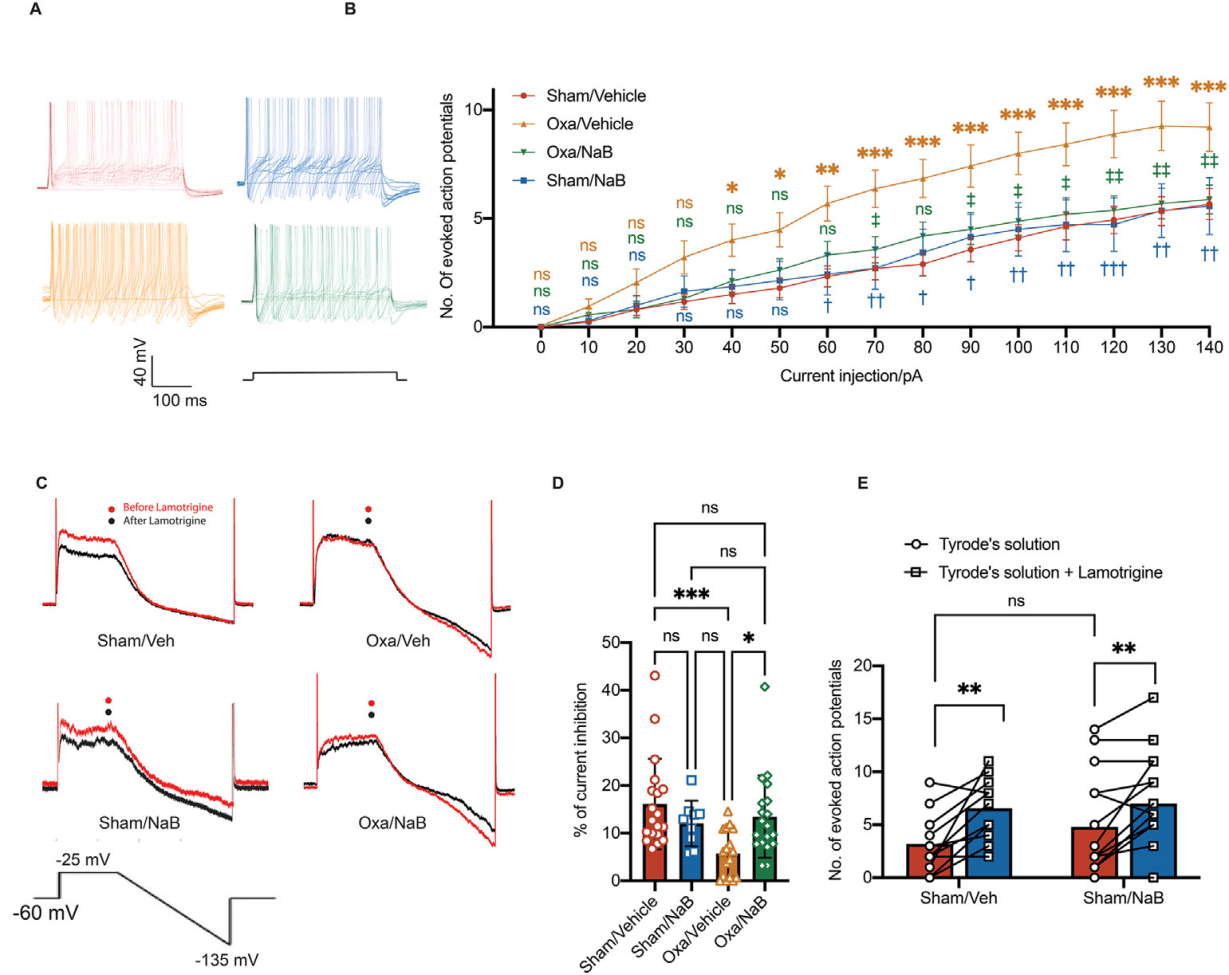
**Sodium butyrate repressed TRESK-induced hyperexcitability of DRG neurons in oxaliplatin-treated mice.** **A**, Representative current clamp recording action potentials (APs) traces of DRG neurons isolated from male mice followed by vehicle treatment (Sham/Veh), male mice received sodium butyrate only (Sham/NaB), male mice received oxaliplatin followed by vehicle treatment (Oxa/Veh), or male mice received oxaliplatin followed by sodium butyrate treatment (Oxa/NaB) on day 14. **B**, Quantification of AP firing numbers per step in the DRG neurons isolated from male mice in the 4 groups. Two-way ANOVA followed by Tukey's multiple comparisons test, *n* ​= ​30 ​cells in Sham/Veh group, *n* ​= ​15 ​cells in Sham/NaB group, *n* ​= ​20 ​cells in Oxa/Veh group, *n* ​= ​30 ​cells in Oxa/NaB group, at least 5 male mice were included in each group. ns, non-significant. Sham/Veh vs Oxa/Veh, ∗*p* ​< ​0.05, ∗∗*p* ​< ​0.01, ∗∗∗*p* ​< ​0.001. Oxa vs NaB, †*p* ​< ​0.05, ††*p* ​< ​0.01. Mean ​± ​S.E.M. **C**, *Top*, representative current traces form small sized (<25 ​μm in diameter) DRG neurons isolated from male mice in Sham/Veh, Sham/NaB, Oxa/Veh or Oxa/NaB groups before and after the application of 45 ​μM lamotrigine. *Bottom*, the voltage protocol adopted to record persistent outward currents. **D**, Quantification of the percentage of lamotrigine-sensitive persistent K^+^ currents in small sized DRG neurons. One-way ANOVA followed by Tukey's multiple comparisons test, *n* ​= ​11–15 DRG neurons from 3 to 4 male mice in each group. ns, non-significant, ∗*p* ​< ​0.05, ∗∗∗*p* ​< ​0.001. **E**, Number of evoked action potentials in sham/vehicle group and sham/NaB group before and after perfusion with Lamotrigine at 90 ​pA current injection. Paired *t*-tests between before and after lamotrigine application. Unpaired *t*-tests between Sham/Veh and Sham/NaB groups. *n* ​= ​8–9 ​cells per group. ∗∗*p* ​< ​0.01.

Twik-related-spinal cord K^+^ (TRESK) channel, encoded by *Kcnk18*, was one of the 4 major types of K^+^ channels highly expressed in cell bodies of small (diameter 10–16 ​μm) to medium (diameter 17–25 ​μm) DRGs from human and mice [42]. *In vivo* knockdown of TRESK channel decreased the paw withdrawal threshold to mechanical painful stimuli, implicating TRESK channels in pain hypersensitivity [35]. In the mice model of oxaliplatin-induced neuropathy, repressed TRESK expression was restored in the mice supplemented with NaB (Fig. 5). We next asked whether NaB treatment altered lumbar DRG excitability via regulating TRESK current. To dissect the effect of NaB on lumbar DRG excitability, we isolated lumbar DRG neurons from mice receiving vehicle or oxaliplatin with or without NaB treatment for whole-cell current clamp recording of action potentials. DRG neurons extracted from mice receiveing oxaliplatin displayed an increased action potential frequencies induced by current injection of 40–140 ​pA, comparing to mice from control group (*p* ​< ​0.001, Fig. 6A and B). NaB treatment completely abolished the oxaliplatin-induced increase in action potential frequency at current injections of 70, 90–140 ​pA (Fig. 6A and B).

We further dissected the effect of NaB on TRESK-mediated currents by measuring the percentage of 45 ​μM lamotrigine-sensitive outward currents. Compared to the control animals, less outward current was inhibited by lamotrigine in the oxaliplatin-treated animals (Veh VS Oxa, 11.9 ​± ​5.0 ​% vs 6.4 ​± ​5.1 ​%, *p* ​< ​0.05, Fig. 6C and D). A higher fraction of outward currents was inhibited by lamotrigine after NaB treatment in the oxaliplatin-treated animals (oxa/vehicle vs oxa/NaB, 6.4 ​± ​5.1 ​%, vs 12.0 ​± ​6.2 ​%, *p* ​< ​0.05, Fig. 6C and D). We measured the number of evoked action potential in sham/vehicle group and sham/NaB group before and after perfusion with Lamotrigine. Our results showed that more action potentials were evoked by 90 ​pA current injection after lamotrigine challenge in both groups (Fig. 6E), while the number of action potentials remained similar in these two groups. These suggested that NaB does not have an unspecific effect on general excitability, and the reduction in DRG hyperexcitability in oxaliplatin-treated mice is due to TRESK. These data suggested that NaB treatment reversed the enhanced DRG neuronal excitability and increased the magnitudes of TRESK current, leading to an increase in the total background K^+^ current, in the oxaliplatin-mediated CIPN model.

## Discussion

This study provided the evidence to support that daily NaB treatment protected against oxaliplatin-induced pain hypersensitivities to mechanical and cold stimuli in mice. Functionally, NaB reduced oxaliplatin-induced lumbar DRG hyperexcitability via regulating TRESK currents. We proposed that NaB directly targeted sensory neurons resided in DRG tissues and enhanced H3K9/H3K14 acetylation for restoring K^+^ expression. The HDAC inhibitory property of NaB may explain its protection against oxaliplatin-induced neuropathic pain.

Our mice model of CIPN with oxaliplatin exhibited an increased mechanical and cold allodynia after repeated doses that was clinically relevant to human chemotherapy (Fig. 1, Fig. 3B and C), in consistent with our previous study in rat, reproducing oxaliplatin-induced neurological symptoms with chronic oxaliplatin neuropathy in human [9,43]. We found up-regulations of *Il-1β*, *Il-6* and *Tnf-α* in the DRGs of oxaliplatin-treated mice 14 days after the first dose (Fig. 1D–F). These pro-inflammatory cytokines are well-known mediators involved in multiple animal models of pain, including oxaliplatin-induced peripheral neuropathy [44,45]. We confirmed, at behavioral and molecular levels, the establishment of our mice model could be used for testing novel candidates in CIPN-related pain conditions.

HDAC is a key epigenetic modulator that has been reported as a molecular target at different anatomical levels in the pain pathway for analgesic development. Differential expressions of HDACs were found at DRGs, spinal cord and pain-related brain regions in different rodent models of pain. For example, after injection of Complete Freund's Adjuvant (CFA) into the ankle joint of rat, HDAC1 and HDAC5 were upregulated within the superficial spinal cord dorsal horn [46]. In the spared nerve injury-induced model of neuropathic pain, HDAC2 and HDAC5 were down-regulated in the superficial dorsal horn but up-regulated in the injured DRGs [46,47]. HDAC1, HDAC2 and HDAC4 were up-regulated in the rat DRGs after spinal nerve ligation [47]. Previous studies showed that a single dose of oxaliplatin induced an increase in HDAC3 expression in DRG neurons while repeated doses failed to induce any transcriptional changes of class 1 HDACs (HDAC1, HDAC2 and HDAC3) in RNAseq analysis, although the class I HDAC inhibitor, MS-275, prevented oxaliplatin-induced neuropathy and reduced its related pain hypersensitivity [14,15]. We here explored differential expression of HDACs by rt-qPCR in DRG tissues at day 14 after repeated oxaliplatin injections. As opposed to the RNAseq study, we identified HDAC1, a class I HDACs, was transiently up-regulated in DRG neuronal nuclei at mRNA and protein levels in the oxaliplatin-induced peripheral neuropathy model (Fig. 2) [15]. We further demonstrated NaB enhanced global histone acetylation in DRG neurons as a class I/IIa HDACI (Fig. 4). This suggested that oxaliplatin-induced HDAC1 up-regulation and the descending histone acetylation in DRG neurons may also be a direct target of NaB.

Down-regulation of genes encoding K^+^ channels, such as the voltage-dependent K^+^ family (Kv1.1 and Kv4.3) and the K2P subfamily (TRESK, TREK-1, TRAAK) were reported in mice DRGs following oxaliplatin administration. In this study, we reported that *Kcnj9*, *Kcnk18* and *Kcnq2* were down-regulated among other K^+^ channel genes in mice DRG tissues after oxaliplatin administration (Fig. 5A). KCNQ2 downregulation was previously reported in the trigeminal ganglia of oxaliplatin-treated mice, while its potentiator retigabine alleviates trigeminal neuropathic pain. However, our results showed that NaB treatment has no effect on reversing oxaliplatin-induced repression of *Kcnq2*. We further demonstrated NaB treatment reversed the expression of *kcnj9* and *kcnk18* in DRG tissues in mice receiving oxaliplatin (Fig. 5B and C). *Kcnj9* encodes the GIRK3 (Kir3.3) subunit of the G protein-gated inwardly rectifying potassium channel in the DRG and is involved in rat with peripheral nerve injury [48]. Mice with genetic knockout of *Kcnj9* have attenuated analgesic responses [49]. Our study was the first to report the down-regulation of *Kcnj9* in DRG tissues of mice receiving oxaliplatin, while its function on neuronal sensitivity requires further exploration due to the absence of a promising and specific antagonist. *Kcnk18* encodes the TRESK channel and is highly expressed in DRG tissues and constitutes the major component of K^+^ background in sensory neurons [42]. Consistent with previous report [14], *Kcnk18* was down-regulated in the DRG tissues in our oxaliplatin model (Fig. 5A and C). It was also reported that mice lacking TRESK channel presented mechanical and cold allodynia [50]. In this study, we showed that NaB treatment attenuated oxaliplatin-induced pain hypersensitivity, accompanied by the reversal of the enhancement of DRG neuronal excitability and the increase in total background K^+^ current in the oxaliplatin model (Fig. 6). These results supported that the pain-alleviating effect of NaB could be at least partially mediated through *Kcnk18* modulation. The transcription factor neuron-restrictive silence factor (NRSF) and its epigenetic co-repressors HDACs were involved in oxaliplatin-mediated K^+^ channel down-regulation and the anti-nociceptive effect of NRSF knockdown was reproduced in mice with Class 1 HDAC inhibition [14]. Whether NRSF or HDAC1 dysregulation could directly influence TRESK channels expression are uncertain. Further mechanistic investigation on whether NaB reversed TRESK channel expression thus reversing oxaliplatin-induced primary afferent hypersensitivity via HDAC inhibition would substantiate the potential use of NaB in patients with oxaliplatin-mediated chemotherapy.

A majority of current studies have demonstrated HDAC activities are altered in microglia and astrocytes in rodent's model of pain, but whether it directly acts on DRG neurons is unknown. In this study, we showed that NaB directly targeted primary sensory neurons (Fig. 2, Fig. 4, Fig. 6), providing evidence that neuronal HDAC activities are involved in the ascending neuronal pathway of pain upon oxaliplatin challenge. We propose that NaB provides HDAC inhibitory effect in addition to the well-known anti-inflammatory actions on neuropathic pain. We tested the effect of intraperitoneal injection of NaB to demonstrate its systemic effect on oxaliplatin-induced pain. Oral administration of NaB attenuates neuropathic pain symptoms [24]. After entering circulatory system, NaB crosses the blood-brain barrier. We could not exclude gut absorption of NaB may also target non-neuronal tissues or other anatomical components, such as spinal cord or brain regions involved in the pain pathway.

**Fig. S3:**
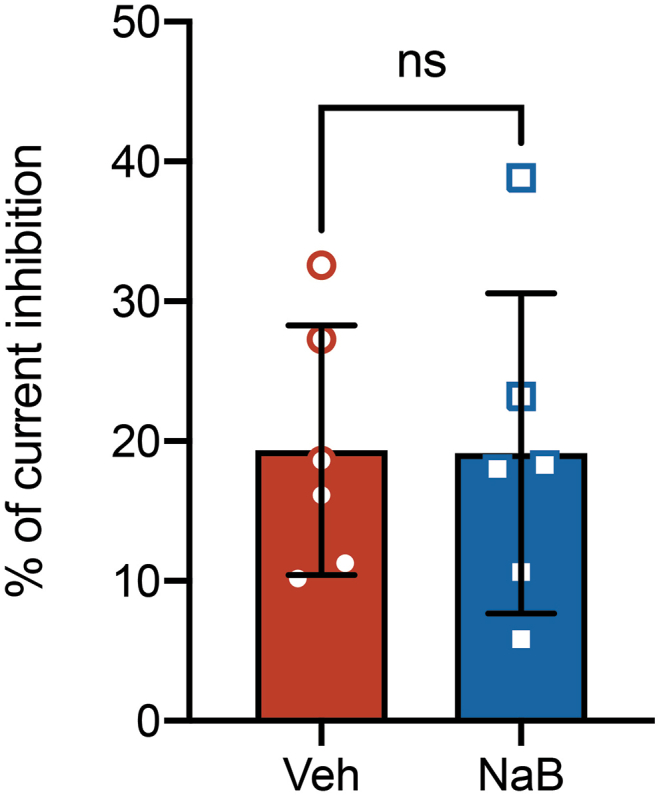
**Acute butyrate treatment did not affect outward current induced inhibited by lamotrigine.** Outward potassium current in DRG neurons inhibited by lamotrigine was measured with electrophysiology. After 10 min of acute butyrate application via perfusion, outward current inhibited by lamotrigine was measured again. Percentage of current inhibition of 6 DRG small-diameter neurons was reported. Unpaired t-test. *ns*: non-significant.

**Fig. S4:**
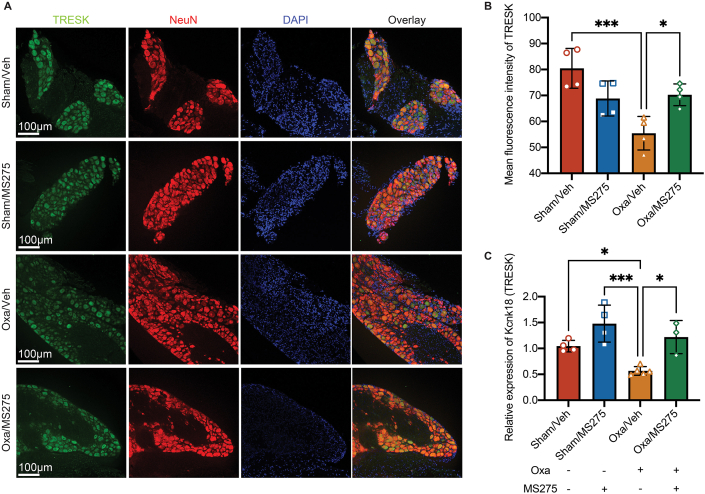
**MS275administration restored oxaliplatin-induced TRESK repression.** **A**, Representative immunofluorescence of TRESK (green), NeuN (red) and DAPI (blue) in L3-L6 DRG tissues collected at day 14 post first dose of oxaliplatin. Male mice were treated with sham or oxaliplatin injection followed by oral gavage of MS-275. **B**. Quantification of mean fluorescence intensity of TRESK in NeuN-positive cells. One-way ANOVA followed by Tukey's multiple comparisons test, *n* ​= ​at least 4 male mice per group. Data was presented as a mean quantification of 3–4 DRG slices. ns, non-significant, ∗∗*p* ​< ​0.01, ∗∗∗*p* ​< ​0.001. **C**, Expression levels of TRESK encoding gene (*kcnk18*) in DRG tissues of mice receiving oxaliplatin (Oxa) or vehicle (Veh) with or withour MS275. One-way ANOVA followed by Tukey's multiple comparisons test, *n* ​= ​3–5 male mice per group. *p*-values not shown were of no significant difference, ∗*p* ​< ​0.05, ∗∗*p* ​< ​0.01.

Butyrate can signal through G protein-coupled receptors (GPCRs) [51]. By acting through GPR41 and GPR43, butyrate could enhance intracellular free cytosolic Ca^2^^+^ [52]. In neurons, sodium butyrate was demonstrated to activate the GRP41/Gβγ/PI3K/Akt pathway and reduce neuronal apoptosis in a stroke model [53]. In our study, we cannot exclude the possibility that sodium butyrate targets GPCRs directly to influence TRESK current or other ion channel functions, nor other epigenetic independent mechanism. However, global histone acetylation was increase in primary DRG culture treated with butyrate (Fig. 4A). Acute application of sodium butyrate did not further repress outward currents inhibited by lamotrigine in DRGs from naïve mice (Supplementary Fig. 3). In addition, oxaliplatin-induced TRESK reduction was restored by Ms-275 administration in mice, mimicking the effect of sodium butyrate (Supplementary Fig. 4). These data strongly suggested that sodium butyrate restored TRESK current, at least partly, via its HDAC1 inhibitory characteristic.

One of the limitations of the current studies is the lack of information on cell type-specific alterations in the HDAC-TRESK pathway. Advances in single-cell omics have revealed tens of neuronal subtypes that could convert differential stimuli and convey tactile or painful sensations. Future studies aiming at detecting cell type-specific reductions in TRESK may provide insights into the development of specific pain hypersensitivities, such as cold allodynia and tactile allodynia, and reveal the detailed pharmacological actions of NaB.

Novel analgesic against oxaliplatin-induce peripheral neuropathy should relieve pain or improve patient's reported outcomes. At the same time, it should not diminish the anti-tumor efficacy of oxaliplatin. Recent studies showed that NaB may have synergistic effects on anti-tumor functions during chemotherapy. In mice model of CRC, NaB synergized with oxaliplatin to inhibit tumor, possibly via inducing cell apoptosis and inhibiting cell proliferation [54]. The combination of NaB and cisplatin enhanced apoptosis of gastric cancer cells to a greater extent than cisplatin administration alone [55]. Our current study indicated NaB could be a useful analgesic for patients undergoing oxaliplatin-based chemotherapy. People with distal pain tend to have lower circulating short chain fatty acids (butyrate, propionate, acetate and valerate) [56]. Supplementation of NaB may be a possible preventive treatment against oxaliplatin-based chemotherapy.

In conclusion, this study provided evidence that sodium butyrate supplementation is a useful analgesic candidate for the treatment of oxaliplatin-induced peripheral neuropathy-related pain. NaB directly targeted DRG neurons to repress TRESK-induced hypersensitivity via its inhibitory action on HDAC.

## Author Contributions

I.H.T.H. and X.L. were responsible for the conceptualization and design of the study. I.H.T.H. and Y.Z. conducted the experiments, analyzed and interpreted the data. K.L., F.Q., Y.J., Q.L., T.J., and X.Z. finished a part of the experiments. I.H.T.H. wrote the manuscript. T.G., L.Z., W.K.K.W., M.T.V.C, C.J and X.L critically revised the manuscript. M.T.V.C secured the funding for the study. All authors read and approved the submission of this manuscript for publication.

## Declaration of competing interest

The authors declare that they have no known competing financial interests or personal relationships that could have appeared to influence the work reported in this paper.
